# Counseling Messages for Adults with Impaired Fasting Glucose in a Public Mobile Healthcare Program: A Structural Topic Model Analysis Using the IMB Framework

**DOI:** 10.3390/nu18101536

**Published:** 2026-05-12

**Authors:** Sarang Jang, Seulki Son

**Affiliations:** 1 Department of Public Health, Sahmyook University, Seoul 01795, Republic of Korea; srjang@syu.ac.kr; 2 Institute of Health & Environment, Seoul National University, Seoul 08826, Republic of Korea

**Keywords:** mobile health, nutrition counseling, impaired fasting glucose, structural topic modeling, Information–Motivation–Behavioral Skills (IMB) model

## Abstract

**Background/Objectives**: Mobile health (mHealth) interventions are commonly used in public healthcare systems to support chronic disease prevention and self-management. However, limited evidence exists regarding the structural composition and theoretical alignment of counseling message content delivered through such programs. This study aimed to identify the latent content structure of nutrition counseling messages for adults with impaired fasting glucose (IFG) and to evaluate their alignment with the Information–Motivation–Behavioral Skills (IMBs) model. **Methods**: A total of 3130 de-identified nutrition counseling messages from 532 adults with IFG enrolled in a nationwide public mobile healthcare program in South Korea were analyzed. A Structural Topic Model (STM), with program phase (months 1–6) as a prevalence covariate, was applied to identify latent topics and temporal variation in topic prevalence. The resulting topics were independently classified into the three constructs of the IMB model by two researchers, with 90% inter-rater agreement, to assess the theoretical alignment of message content. **Results**: STM identified ten topics, which were classified as information (seven topics, 64.3%), behavioral skills (two topics, 28.5%), and motivation (one topic, 7.2%). The program phase was a significant predictor of prevalence for 9 of the 10 topics, with information surging to 88.7% in Phase 4 due to seasonal food safety messaging. Behavioral skills topics were most prevalent at Phase 1 and Phase 6, whereas motivation remained consistently low throughout the intervention. **Conclusions**: Nutrition counseling messages were heavily weighted toward information delivery, with limited motivational content, suggesting an imbalance in the key components required for behavior change under the IMB framework. Temporal patterns appeared to follow operational or seasonal factors rather than a theory-driven progression. These findings highlight the need for more theory-informed message design in public mobile healthcare programs, with the potential for AI-assisted approaches to enable personalized and adaptive counseling content.

## 1. Introduction

Rapid advances in digital health have enabled the delivery of health services beyond traditional clinical settings. In particular, mobile health (mHealth) platforms facilitate continuous, remote interaction between healthcare providers and individuals, supporting real-time health management and behavior change [1,2,3]. Within this context, mHealth-based counseling has emerged as an important approach for delivering ongoing behavioral support through routine communication.

Among the populations that may benefit from mHealth-based counseling, adults with impaired fasting glucose (IFG) represent a particularly relevant target group. IFG is defined as a fasting plasma glucose level ranging from 100 to 125 mg/dL and represents a typical prediabetic state positioned between normoglycemia and diabetes [4,5]. This stage is widely regarded as a critical window for intervention, during which dietary management plays a central role in preventing progression to type 2 diabetes and supporting long-term glycemic control [6,7].

Nutrition counseling messages delivered through mHealth programs represent a key mechanism for providing continuous behavioral support. By incorporating behavior change techniques such as goal setting, feedback, and information provision, these messages can deliver practical dietary guidance, reinforce motivation, and support the development of self-management capacity [8,9,10,11]. Unlike traditional one-time counseling sessions, mHealth-based messaging enables repeated, context-sensitive interactions that may enhance engagement and facilitate sustained behavior change. However, despite their widespread use, there remains limited empirical evidence regarding how nutrition counseling messages are structured and how their content reflects established behavior change frameworks.

To provide a theoretical basis for evaluating the content of nutrition counseling messages, this study applies the Information–Motivation–Behavioral Skills (IMB) model proposed by Fisher and Fisher (1992) [12]. The IMB model posits that effective behavior change is shaped by three interrelated components: (1) accurate and relevant information necessary for performing the target behavior, (2) personal and social motivation to engage in that behavior, and (3) behavioral skills required to translate intention into action [13,14]. The model emphasizes that information alone is insufficient to produce sustained behavior change and has been widely used to guide the design of interventions that integrate informational, motivational, and skill-based components, particularly in mobile healthcare settings [15,16]. In this study, the IMB model provides a structured framework for examining whether counseling messages in mHealth settings adequately incorporate these key components.

The IMB framework has been applied across a range of chronic disease contexts, including diabetes self-management and glycemic control, enabling the identification of gaps in intervention design and supporting the development of more effective health communication strategies [17,18]. Applying this framework to nutrition counseling messages allows for a more systematic evaluation of message content beyond thematic categorization, particularly in terms of its alignment with behavioral determinants of self-management [15,17,19].

To operationalize this approach, the present study employs Structural Topic Modeling (STM), which enables the identification of latent thematic structures within large-scale unstructured text while incorporating document-level covariates directly into the model [20,21]. Unlike Latent Dirichlet Allocation (LDA), STM allows us to examine how topic prevalence varies according to contextual factors [22,23]. In this study, this feature is used to analyze changes in topic distribution across intervention months, with the aim of exploring how the content of nutrition counseling messages evolves over the course of the six-month program.

Previous studies on mobile healthcare have primarily focused on improvements in quantitative health outcomes, such as changes in blood glucose levels or body weight following intervention [24,25,26,27]. Although some studies have explored the relationships between message delivery frequency, user engagement, and intervention content, relatively few have systematically examined large-scale text data generated during counseling processes using established theoretical frameworks such as behavior change models [28,29].

This study makes three key contributions. First, rather than relying on literature-based or experimental data alone, it analyzes real-world nutrition counseling log data generated within a public health center-based mobile healthcare program, thereby providing an empirical perspective grounded in routine practice. Second, by applying STM, a contemporary text-mining approach, this study captures the dynamic changes in counseling topics over time by incorporating the intervention period as a temporal covariate. Third, by linking the identified topics to the components of the IMB model, the study examines the extent to which counseling messages in public mHealth services reflect key informational, motivational, and behavioral components relevant to behavior change and offers insights to inform the development of data-driven, tailored intervention strategies.

The aim of this study was to identify the latent content structure of nutrition counseling messages delivered through a public mobile healthcare program for adults with IFG. First, it sought to extract the main topics embedded in the counseling messages using STM. Second, it aimed to map the identified topics onto the IMB model to assess the behavioral orientation of message content. Third, it examined temporal changes in topic proportions over the course of the intervention.

## 2. Methods

### 2.1. Study Design

This study employed a retrospective content analysis of de-identified secondary text message data generated within a public health center-based mobile healthcare program in South Korea. The analysis aimed to identify the thematic structure of counseling messages and examine how the message content evolved over the six-month intervention period. An STM [21], an unsupervised probabilistic approach that identifies latent topics based on word co-occurrence patterns, was applied. Each individual message was treated as a single document for modeling purposes, and the program phase was incorporated as a document-level covariate to model temporal variation in topic prevalence, with each phase corresponding to one calendar month out of a six-month period. The resulting topics were interpreted and mapped onto the IMB model to contextualize the identified themes within an established theoretical framework of health behavior change.

### 2.2. Study Setting

The data for this study were derived from a nationwide public mobile healthcare program operated by public health centers in South Korea. The program, supported by the Korea Health Promotion Institute, provides health monitoring, personalized counseling, and behavioral tracking services through a mobile application to community-dwelling adults at risk of chronic disease. Nutrition counseling messages were composed individually by registered dietitians employed at each participating public health center. Messages were not prompted by specific questions from participants; instead, dietitians reviewed each participant’s dietary records and health monitoring data uploaded through the mobile application and composed personalized monthly counseling messages based on this information. Messages were delivered to participants through the program’s mobile application interface. The present study focused on participants identified as having IFG. A total of 3192 nutrition counseling messages sent between 2019 and 2020 were obtained for analysis.

### 2.3. Eligibility of the Message Dataset

From a total of 1509 participants enrolled in the nationwide public health center mobile healthcare program between 2019 and 2020, 532 individuals with IFG were selected. A total of 3192 nutrition counseling messages were obtained for analysis. Only messages containing nutrition counseling content composed by a registered dietitian and delivered within the six-month intervention period were included; system-generated notifications and administrative messages were excluded. Duplicate messages sent to the same participant within the same month, or those that yielded no tokens after preprocessing, such as template-only messages, were also excluded.

### 2.4. Text Preprocessing

All messages were preprocessed using the Kiwi morphological analyzer (Korean Intelligent Word-based Information Extractor) [30]. Non-Korean characters, special symbols, and numbers unrelated to the dietary context were removed. Stopwords, place names, and days of the week were also removed because they carried minimal analytic value. Compound terms frequently used in nutrition counseling, such as sigyiseomyu (dietary fiber) and sigsailgi (food diary), were consolidated into single semantic units using bigram collocation rules.

Tokenization included nouns, adjectives, and verbs, as health counseling messages fundamentally extend beyond the delivery of health knowledge to convey attitudinal and action-directed language. During preliminary testing, alternative strategies retaining only nouns or only nouns and adjectives were compared, but verbs proved essential for capturing action-oriented content such as dietary practice strategies and self-management instructions.

### 2.5. Structural Topic Model

STM extends the LDA framework [31] by allowing document-level covariates to influence topic proportions [20]. Whereas LDA assumes that each document is a mixture of latent topics defined by word probability distributions, STM also models the relationship between document metadata and topic prevalence, enabling researchers to estimate how contextual factors shape the thematic structure of a corpus [20]. In the present study, program phase (months 1–6) was specified as a prevalence covariate, allowing the model to estimate how the composition of counseling content shifted over the course of the intervention.

The optimal number of topics (K) was determined using the searchK() function in the stm R package by evaluating held-out likelihood, semantic coherence, residuals, and lower bound across K = 2–15. Based on a combined assessment of these indicators, K = 10 was selected because it offered a balance between model fit and topic interpretability (Supplementary Figure S1).

Each topic was characterized using the top 15 words ranked by highest probability and by FREX (Frequency and Exclusivity). Highest-probability words provide an intuitive overview of a topic’s general content, but because high-frequency words may appear across multiple topics, FREX was used as a complementary indicator to identify words that are both frequent within and exclusive to a given topic [20]. Two independent researchers with expertise in health behavior theory reviewed both word lists and assigned a descriptive label to each topic. Disagreements were resolved through structured discussion; in cases where consensus could not be reached, a third reviewer made the final determination. All analyses were conducted using R version 4.5.2 with the stm package version 1.3.8.

### 2.6. IMB-Based Interpretation

Each of the ten topics identified by the STM were classified into one of the three constructs of the IMB model. The classification criteria were as follows: topics were categorized as information if they primarily conveyed diet-related factual knowledge, such as nutrient content, physiological mechanisms, or evidence-based dietary guidelines; as motivation if they were dominated by normative expressions, relational encouragement, or attitudinal language aimed at strengthening behavioral intention; and as behavioral skills if they centered on actionable strategies, self-monitoring techniques, or app-based skill development for dietary self-management.

Two researchers with expertise in health behavior theory independently classified all ten topics. Inter-rater agreement was evaluated using percent agreement. Disagreements were resolved through structured discussion; in cases where consensus could not be reached, a third reviewer made the final determination.

### 2.7. Ethical Considerations

This study used de-identified secondary data provided through a formal data use agreement with the Korea Health Promotion Institute. The study was approved by the Institutional Review Board of Wonkwang University (approval number: WKIRB-202111-SB-088). Given the retrospective nature of the study and the use of de-identified data, the requirement for informed consent was waived by the IRB.

## 3. Results

### 3.1. Characteristics of the Message Corpus

The final analytic corpus consisted of 3192 nutrition counseling messages from 532 participants across six program phases. Of these, 62 messages were excluded because they contained no extractable tokens (nouns, adjectives, or verbs) after preprocessing, resulting in a total of 3130 messages that were suitable for analysis. The number of tokens per message ranged from 3 to 289 (median = 49, mean = 57.1). Of the 532 participants, 300 (56.4%) achieved normalization of fasting blood glucose levels by the end of the six-month intervention.

### 3.2. Topics Identified by Structural Topic Modeling

STM analysis identified ten latent topics within the corpus of 3130 nutrition counseling messages. Table 1 presents each topic with its descriptive label, corpus proportion, and representative keywords. The largest topic was T9 (summer food safety, 14.61%), followed by T3 (mobile healthcare program use, 14.41%) and T10 (food diary and self-monitoring, 14.05%). These three topics alone accounted for 43.1% of the total corpus, while the remaining seven topics comprised the other 56.9%. The smallest topic was T4 (physiological mechanisms and nutrients, 4.59%). Collectively, the ten topics covered a broad range of counseling content, from nutrient-specific knowledge and seasonal health guidance to behavioral self-management strategies and mobile healthcare program guidance.

### 3.3. Classification of Topics According to the IMB Framework

The ten topics were classified into the three constructs of the IMB model by two independent researchers, who reached initial agreement on 9 of 10 topics (90%). The one disagreement was resolved through discussion. Table 2 summarizes the distribution of topics by IMB construct.

Seven topics were classified as information, accounting for 64.3% of the total corpus. These topics covered a broad range of diet-related factual content, including nutrient functions, physiological mechanisms, and seasonal health guidance. Two topics were classified as behavioral skills, accounting for 28.5%, and both centered on self-monitoring practices through food diary use and mobile healthcare application features. Only one topic was classified as motivation, representing 7.2% of the corpus; motivational content was primarily expressed through normative and relational language rather than direct motivational appeals.

### 3.4. Temporal Changes in Topic Prevalence

**Figure 1:**
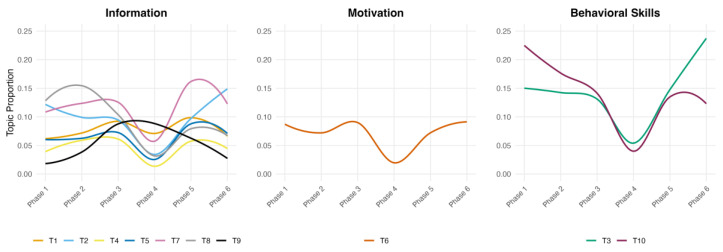
Topic prevalence by IMB construct over the six-month intervention period.

Figure 1 presents the temporal trajectories of topic prevalence across the six program phases, organized by IMB construct. Regression analysis using the estimateEffect() function with natural splines confirmed that program phase was a statistically significant predictor of topic prevalence for 9 of the 10 topics.

Among the information topics, T9 (summer food safety) showed the strongest phase effect, rising sharply from 1.8% at Phase 1 to 65.4% at Phase 4 (*p* < 0.001), thereby dominating message content during this period. T8 (low-sodium diet) exhibited a consistent decline across all spline terms (all *p* < 0.001), decreasing from 12.8% at Phase 1 to 3.2% at Phase 4 before recovering to 7.9% at Phase 5. T7 (micronutrients and dietary fiber) was the only topic that remained relatively stable, with no significant phase effect on individual spline terms (*p* > 0.05).

The single motivation topic, T6 (normative self-management), showed a significant decline during the mid-intervention period (*p* < 0.001), decreasing from 8.7% at Phase 1 to 2.0% at Phase 4 before returning to 9.1% by Phase 6.

Within behavioral skills, T10 (food diary and self-monitoring) showed significantly negative coefficients across all spline terms (all *p* < 0.001), declining from 22.5% at Phase 1 to 4.0% at Phase 4. T3 (mobile healthcare program use) exhibited a significant nonlinear trajectory (*p* < 0.001), remaining stable through Phase 3, dipping to 5.4% at Phase 4, and then rising to 23.7% at Phase 6, reaching the highest proportion among all topics in the final phase.

At the IMB construct level, information accounted for 53.7–64.5% of message content across Phases 1–3, 5, and 6, but surged to 88.7% at Phase 4 due to the dominance of T9. behavioral skills showed the inverse pattern, declining from 37.5% at Phase 1 to 9.4% at Phase 4 before recovering to 36.0% at Phase 6. Motivation remained consistently low across all phases, ranging from 2.0% to 9.1%.

## 4. Discussion

This study examined nutrition counseling messages delivered through a public mobile healthcare program for adults with IFG using STM and the IMB model. The findings showed that informational content accounted for the largest share of messages (64.3%), whereas motivational components were relatively limited (7.2%). Behavioral skills represented a substantial proportion (28.5%), but were primarily concentrated on self-monitoring activities. Temporal analysis revealed significant variation in message content across program phases, with certain topics, such as seasonal food safety, showing sharp increases during the mid-intervention period, while self-management-related messages became more prominent toward the end of the program. Taken together, these results suggest that current counseling messages in public mobile healthcare settings do not reflect a balanced integration of the core components proposed by the IMB model.

The predominance of information-oriented messages observed in this study appears to reflect the operational approach of public mobile healthcare programs, which tend to deliver standardized health information to a large number of participants [32]. However, information alone is rarely sufficient to sustain health behavior change; rather, behavior change is more likely to occur when information is accompanied by both motivation and behavioral skills [33,34]. While prior mHealth studies have largely focused on intervention effects on health outcomes, relatively few have examined the structure of counseling message content or its alignment with behavior change theories [35,36]. In addition, text-mining studies in health communication have mainly focused on social media or online information, paying limited attention to counseling data generated within public health programs [37,38]. By linking public mobile healthcare counseling messages to a behavior change framework, this study helps address these gaps while also highlighting the limitations of standardized, information-heavy messaging and the need for more tailored interventions [32,35]. These findings offer important insights into how the composition of message content in public mobile healthcare programs may influence the mechanisms underlying behavior change.

However, it should be noted that participant motivation was not directly assessed in this study; therefore, it remains unclear whether the relatively low proportion of motivational message content reflects an unmet need or was appropriate given participants’ existing motivation levels. Given the increasing accessibility of health information through digital sources, mHealth counseling interactions may represent a particularly valuable opportunity to reinforce motivation beyond information provision alone. Future studies should consider incorporating validated motivational assessment tools to evaluate changes in participant motivation over the course of the intervention, which would enable a more precise examination of whether motivational message content is aligned with participants’ actual needs at each stage of the program.

In this study, the observed shifts in message content over the course of the intervention appear to reflect program strategies that emphasize specific health behaviors at different time points. The increase in low-sodium diet-related messages during the fourth month may be interpreted as a result of targeted goal-setting efforts during that period, while the rise in self-management messages toward the end of the program likely represents an attempt to strengthen participants’ capacity for autonomous health management [39]. However, from an overall perspective, the progression from information provision to motivation enhancement and behavioral skill development was not implemented in a sufficiently structured manner. Behavior change theories generally recommend a phased messaging approach, with basic information delivered in the early stage, motivation reinforced through goal setting and feedback in the middle stage, and self-monitoring along with behavioral skills emphasized in the later stage to support long-term change [28,39]. This stepwise pattern is also consistent with stage-based approaches in behavior change theory, which emphasize the importance of aligning intervention strategies with the evolving needs of participants over time [40,41].

Notably, the behavioral skill-related messages identified in the present study were predominantly focused on self-monitoring and app-based program use, suggesting that current messaging may support the initiation of dietary behavior change but may be insufficient for its long-term maintenance. Evidence indicates that sustaining dietary behavior change requires competencies beyond self-monitoring, such as habit formation, coping with adherence barriers, and managing high-risk situations [42,43,44,45]. Incorporating such content into future mHealth message design may enhance the long-term effectiveness of public mobile healthcare programs.

While the present study was not designed to develop or evaluate an intervention framework, the patterns observed in message composition may offer useful implications for future message design. Within the context of the IMB model and established behavior change theories, the findings suggest that message delivery could be more systematically structured, with a focus on motivational enhancement in the early stage, behavioral skill development in the middle stage, and maintenance and relapse prevention in the later stage. This perspective is consistent with phase-based approaches proposed in prior behavior change research [28,39]. These implications are derived from the interpretation of the information-dominant structure identified in this study, the relatively low proportion of motivational content, and the temporal variation in message composition influenced by operational and seasonal factors. Taken together, the findings suggest a need to more deliberately strengthen motivational and behavioral skill components in future mHealth programs. This may involve developing phase-specific message guidelines for counselors and incorporating individual health records into message planning to support more adaptive communication.

Unlike private sector mHealth initiatives, public programs are not subject to market-driven content optimization, making systematic analysis of message content particularly important for identifying gaps between delivered content and evidence-based behavior change frameworks and for informing policy-level improvements in program design. Designing messages for public mobile healthcare programs requires a balanced integration of information, motivation, and behavioral skills, as this combination is essential for effectively supporting health behavior change [46,47]. Tailored messaging strategies that draw on participants’ health data and behavioral records can enhance user engagement and intervention effectiveness, and adjusting message content according to individual health status or level of participation is strongly recommended [46,48].

Beyond tailoring content to individual health status, a critical consideration in the design of public mHealth messaging is the substantial heterogeneity among participants in terms of health literacy, educational attainment, and socioeconomic background [49]. The effectiveness of information-heavy messaging is likely to vary considerably across these dimensions, with individuals with lower health literacy potentially receiving less benefit from clinically detailed nutritional content. Future program designs should account for these individual-level factors through adaptive message tailoring informed by participant profiles. In this regard, large language models and generative AI tools may eventually offer scalable approaches to producing literacy-adapted, personalized messages [50]; however, rigorous evaluation of such approaches in public health settings remains an important area for future research.

Advances in artificial intelligence and natural language processing may offer potential for automatically analyzing message structures and developing systems that generate counseling messages aligned with individual behavior patterns. Although such applications remain largely exploratory in the context of public mHealth programs, they represent a promising direction for improving both the efficiency and scalability of mobile health services [51,52]. In addition, refining messages through user-centered design and iterative feedback processes is important for improving cultural relevance and comprehensibility, particularly among low-income or low-health-literacy populations [53,54]. Text-based messaging offers a cost-effective and widely accessible approach, and its effectiveness is enhanced when it incorporates theory-based techniques such as reinforcement, motivation, and goal setting [46,55].

A key strength of this study is that it is based on counseling messages generated within an actual public mobile healthcare program, providing a high level of real-world relevance. The analysis draws on a substantial dataset of 3192 messages, allowing an empirical examination of message content structure. In addition, the use of Structural Topic Modeling enabled simultaneous analysis of both thematic structure and temporal patterns in message content.

Several limitations should also be noted. First, this study relied on secondary data generated during program operation and did not include information on whether participants actually read the messages or the extent of their engagement. Specifically, it was not possible to assess message receipt or frequency of exposure, nor to examine the relationship between message exposure and behavioral responses. However, given that participants attended in-person monitoring visits at public health centers approximately every two months, it is plausible that they were exposed to and aware of the messages to some extent. In addition, the study did not include direct measures of dietary behavior or adherence, which limits our ability to examine the relationship between message content and actual behavior change. These aspects fall outside the scope of the present content-focused analysis and should be addressed in future research. Second, the process of mapping topics to IMB categories may have involved some degree of subjective interpretation. Third, the messages analyzed were drawn from a program conducted between 2019 and 2020, and program delivery approaches may have evolved since then. Finally, as the findings are grounded in the context of the Korean public health system and its dietary culture, caution is warranted when applying these results to other settings. Nevertheless, the observed imbalance between information-heavy and motivation- or skill-focused messaging may reflect a broader pattern in public mHealth programs and may offer useful considerations for other contexts, including the need to examine whether messages are overly information-focused and whether sufficient attention is given to motivational content and behavioral skill development.

## 5. Conclusions

This analysis of nutrition counseling messages from a public mobile healthcare program suggests that the content is heavily weighted toward information, while motivational elements remain relatively limited within the IMB framework. In other words, the overall structure of messaging appears unbalanced in terms of the key components required for behavior change. Over time, message content seemed to follow operational or seasonal patterns rather than a theory-driven sequence. The expected progression, from providing information to strengthening motivation and then supporting behavioral skills, was not consistently observed throughout the intervention period. Taken together, these patterns indicate that current public mHealth messaging practices are not fully aligned with established behavior change frameworks. Improving effectiveness will likely require a more deliberate structuring of messages, with a clearer focus on how informational, motivational, and behavioral elements are combined and delivered over time. There is also growing potential to improve this process through data-driven approaches. In particular, AI-assisted systems may potentially support the development of messages that better reflect individual health status and behavioral patterns, allowing for more responsive and personalized communication. Future research is needed to evaluate the feasibility and effectiveness of such approaches. From a practical perspective, this study highlights the need to rethink how messaging is designed within public digital health programs. Moving toward more theory-informed and adaptive approaches may help strengthen engagement and support sustained self-management. Future work should examine whether more systematically structured messaging, grounded in behavior change theory, leads to measurable improvements in health behaviors and how such approaches can be implemented at scale within public mHealth systems. Future studies employing experimental or quasi-experimental designs, in which message types are systematically varied, would be better positioned to establish relationships between message content and fasting glucose normalization and provide more direct evidence of the effectiveness of theory-informed message design.

## Figures and Tables

**Table 1:** Topics identified by structural topic model (K = 10).

Topic	Label	Proportion ^1^ (%)	Highest Probability	FREX
T1	Energy Balance and Seasonal Health	7.70	Energy, meal portion, immunity, COVID-19, lean meat, hyperlipidemia	Energy, COVID-19, hyperlipidemia, cause, fast food, yogurt
T2	Dietary Lifestyle and Feedback	9.96	intake, dietary life, dairy, greasy, contain, examine	dietary life, dairy, breakfast, drinking occasion, feedback, meat and fish
T3	Mobile Healthcare Program Use	14.41	mobile, healthcare, dietitian, health center, health management, return	mobile, healthcare, health center, coordinator, enter into, pass by
T4	Physiological Mechanisms and Nutrients	4.59	consist of, flour, insulin, hormone, decrease, curious	flour, amino acid, increase, heavy metal, kalguksu, cataract
T5	Calorie and Weight Management	6.34	calorie, satiety, salad, sweet potato, diet, body fat	calorie, satiety, sweet potato, approach, chicken breast, coffee mix
T6	Normative Self-Management	7.22	carbohydrate, eating habits, consistent, appropriate amount, one week, low-sodium diet	carbohydrate, storage, in one’s mind, makgeolli, report, one night
T7	Micronutrients and Dietary Fiber	11.68	nutrient, dietary fiber, vitamin, cholesterol, rich in, mineral	mineral, fatty acid, antioxidant, omega, alcohol, pork
T8	Low-Sodium Diet	9.44	sodium, seaweed, solid ingredients, eating habits, tomato, banana	sodium, tomato, banana, pickled vegetables, processed food, red pepper paste
T9	Summer Food Safety	14.61	participant, summer, food poisoning, mostly, moderate amount, beverage	summer, food poisoning, beverage, extreme heat, infectious disease
T10	Food Diary and Self-Monitoring	14.05	food diary, protein, multigrain rice, recommended intake, simple, cumbersome	graph, americano, shortcut, bulgogi, similar, page

^1^ Proportion indicates the mean topic prevalence across the corpus.

**Table 2:** Distribution of topics by IMB model construct.

IMB Construct	Combined Proportion (%)	Topics	Representative Keywords
Information	64.3	1, 2, 4, 5, 7, 8, 9	sodium, antioxidant, calorie, carbohydrate, vitamin, insulin, dietary fiber, cholesterol, food poisoning
Motivation	7.2	6	consistent, appropriate amount, eating habits, mindset
Behavioral Skills	28.5	3, 10	mobile, healthcare, food diary, graph, health management, self-monitoring

## Data Availability

The data presented in this study are not publicly available due to privacy and ethical restrictions but are available from the corresponding author on reasonable request.

## Supplementary Materials

The following supporting information can be downloaded at https://www.mdpi.com/article/10.3390/nu18101536/s1, Figure S1. Model Selection Diagnostics for Structural Topic Model.

## Abbreviations

The following abbreviations are used in this manuscript:

IFG	Impaired Fasting Glucose.
IMB	Information–Motivation–Behavioral Skills.
LDA	Latent Dirichlet Allocation.
STM	Structural Topic Modeling.
