# Supplementary material for: Counseling Messages for Adults with Impaired Fasting Glucose in a Public Mobile Healthcare Program: A Structural Topic Model Analysis Using the IMB Framework
Source: Nutrients. 2026 May 12;18(10):1536. doi: 10.3390/nu18101536 (PMC13209786; doi:10.3390/nu18101536)
Supplement: Supplementary file 1 [file nutrients-18-01536-s001.zip › nutrients-4253393-supplementary.pdf]

Supplementary Materials

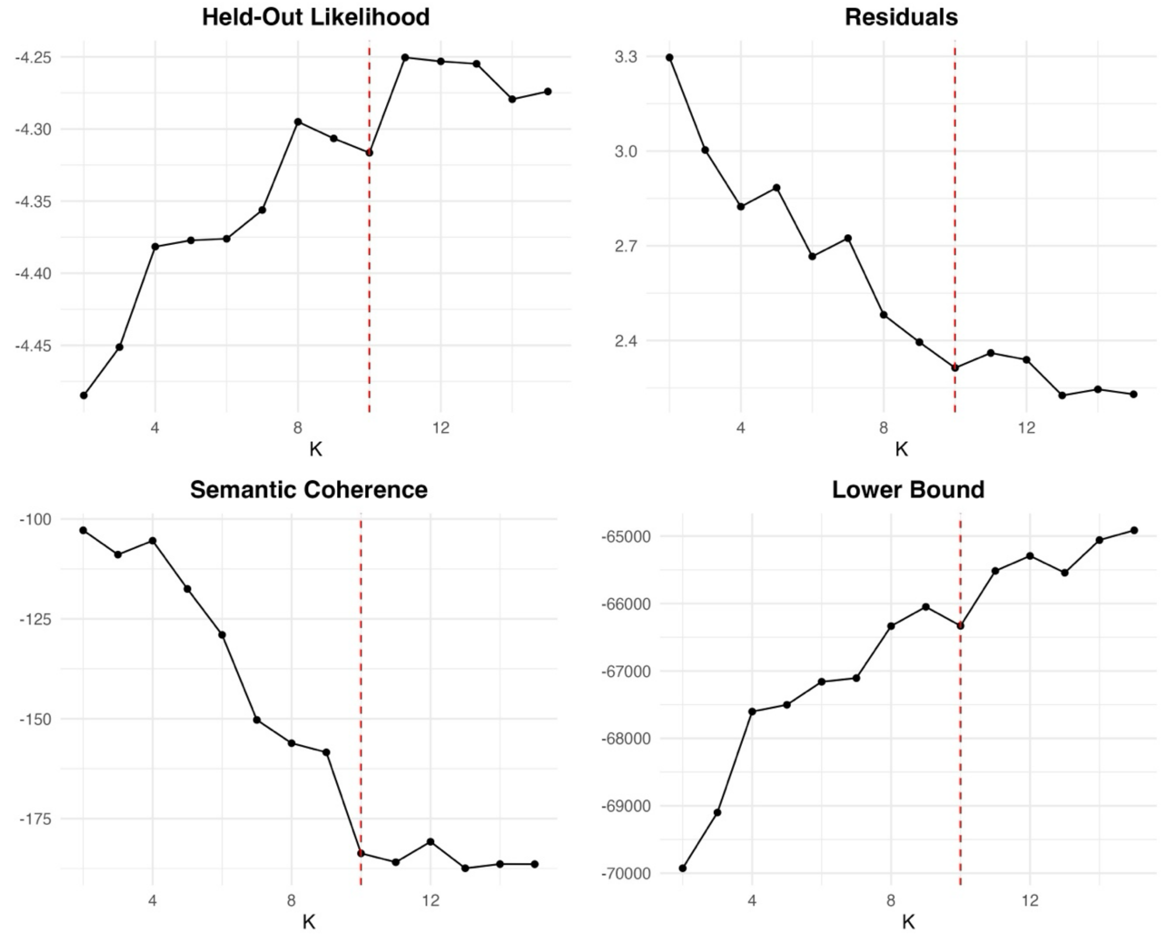

Figure S1. Model Selection Diagnostics for Structural Topic Model

Diagnostic values for topic number selection (K = 2–15). Four indicators, including held-out likelihood, residuals, semantic coherence, and lower bound, were evaluated using the searchK() function in the stm R package. The red dashed line indicates the selected number of topics (K = 10). K = 10 was chosen because it offered a balance between model fit (held-out likelihood and residuals) and topic interpretability (semantic coherence), with diminishing improvements observed beyond this point.
